# Transmembrane potential induced on the internal organelle by a time-varying magnetic field: a model study

**DOI:** 10.1186/1743-0003-7-12

**Published:** 2010-02-20

**Authors:** Hui Ye, Marija Cotic, Eunji E Kang, Michael G Fehlings, Peter L Carlen

**Affiliations:** 1 Toronto Western Research Institute, University Health Network, Toronto, Ontario, M5T 2S8, Canada; 2 Department of Physiology, University of Toronto, Toronto, Ontario, M5S 1A1, Canada; 3 Institute of Biomaterials and Biomedical Engineering, University of Toronto, Toronto, Ontario, M5S 1A1, Canada; 4 Department of Surgery, University of Toronto, Toronto, Ontario, M5S 1A1, Canada

## Abstract

**Background:**

When a cell is exposed to a time-varying magnetic field, this leads to an induced voltage on the cytoplasmic membrane, as well as on the membranes of the internal organelles, such as mitochondria. These potential changes in the organelles could have a significant impact on their functionality. However, a quantitative analysis on the magnetically-induced membrane potential on the internal organelles has not been performed.

**Methods:**

Using a two-shell model, we provided the first analytical solution for the transmembrane potential in the organelle membrane induced by a time-varying magnetic field. We then analyzed factors that impact on the polarization of the organelle, including the frequency of the magnetic field, the presence of the outer cytoplasmic membrane, and electrical and geometrical parameters of the cytoplasmic membrane and the organelle membrane.

**Results:**

The amount of polarization in the organelle was less than its counterpart in the cytoplasmic membrane. This was largely due to the presence of the cell membrane, which "shielded" the internal organelle from excessive polarization by the field. Organelle polarization was largely dependent on the frequency of the magnetic field, and its polarization was not significant under the low frequency band used for transcranial magnetic stimulation (TMS). Both the properties of the cytoplasmic and the organelle membranes affect the polarization of the internal organelle in a frequency-dependent manner.

**Conclusions:**

The work provided a theoretical framework and insights into factors affecting mitochondrial function under time-varying magnetic stimulation, and provided evidence that TMS does not affect normal mitochondrial functionality by altering its membrane potential.

## Background

Time-varying magnetic fields have been used to stimulate neural tissues since the start of 20th century [1]. Today, pulsed magnetic fields are used in stimulating the central nervous system, via a technique named transcranial magnetic stimulation (TMS). TMS is being explored in the treatment of depression [2], seizures [3,4], Parkinson's disease [5], and Alzheimer's disease [6,7]. It also facilitates long-lasting plastic changes induced by motor practice, leading to more remarkable and outlasting clinical gains during recovery from stroke or traumatic brain injury [8].

When exposed to a time-varying magnetic field, the neural tissue is stimulated by an electric current via electromagnetic induction [9], which induces an electrical potential that is superimposed on the resting membrane potential of the cell. The polarization could be controlled by appropriate geometrical positioning of the magnetic coil [10-12]. To investigate the effects of stimulation, theoretical studies have been performed to compute the magnetically induced electric field and potentials in the neuronal tissue, using models that represent nerve fibers [13-18] or cell bodies [19].

Mitochondria are involved in a large range of physiological processes such as supplying cellular energy, signaling, cellular differentiation, cell death, as well as the control of cell cycle and growth [20]. Their large negative membrane potential (-180 mV) in the mitochondrial inner membrane, which is generated by the electron-transport chain, is the main driving force in these regulatory processes [21-23]. Alteration of this large negative membrane potential has been associated with disruption in cellular homeostasis that leads to cell death in aging and many neurological disorders [24-27]. Thus, mitochondria can be a therapeutic target in many neurodegenerative diseases such as Alzheimer's disease and Parkinson's disease.

Two lines of evidences suggest that the physiology of mitochondria could be affected by the magnetic field via its induced transmembrane potential. First, magnetic fields can induce electric fields in the neural tissue, and it has been shown that exposure of a cell to an electrical field could introduce a voltage on the mitochondrial membrane [28]. This induced potential has led to many physiological/pathological changes, such as opening of the mitochondrial permeability transition pore complex [29]. Nanosecond pulsed electric fields (nsPEFs) can affect mitochondrial membrane [30,31], cause calcium release from internal stores [32], and induce mitochondria-dependent apoptosis under severe stress [33,34]. Secondly, there is evidence that magnetic fields could alter several important physiological processes that are related to the mitochondrial membrane potential, including ATP synthesis [35,36], metabolic activities [37,38] and Ca^2+ ^handling [39,40]. An analysis of the mitochondrial membrane potential is of experimental significance in understanding its physiology/pathology under magnetic stimulation.

In this theoretical work, we have provided the first analytical solution for the transmembrane potential in an internal organelle (i.e., mitochondrion) that is induced by a time-varying magnetic field. The model was a two-shell cell structure, with the outer shell representing the cell membrane and the inner shell representing the membrane of an internal organelle. Factors that affect the amount of organelle polarization were investigated by parametric analysis, including field frequency, and properties of the cytoplasmic and organelle membranes. We also estimated to what degree magnetic fields used in TMS practice affect organelle polarization.

## Methods

### Spherical cell and internal organelle model in a time-varying magnetic field

**Figure 1:**
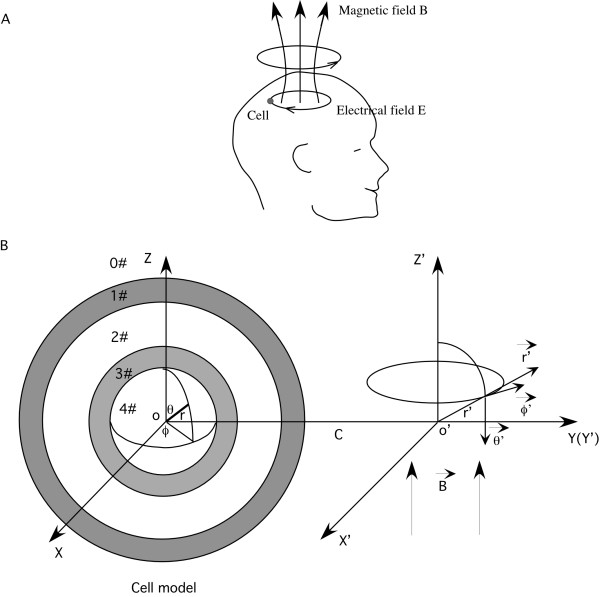
**The model of a spherical cell with a concentric spherical internal organelle**. A. Relative coil and the targeted cell location, and the direction of the magnetically-induced electrical field in the brain. The current flowing in the coil generated a sinusoidally alternating magnetic field, which in turn induced an electric current in the tissue, in the opposite direction. The small circle represented a single neuron in the brain. B. The cell and its internal organelle represented in a spherical coordinates (*r*, *θ*, *ϕ*). The cell includes five homogenous, isotropic regions: the extracellular medium, the cytoplasmic membrane, the cytoplasm, the organelle membrane and the organelle interior The externally applied magnetic field was in cylindrical coordinates (*r*', *ϕ*', *z*'). The axis of the magnetic field overlapped with the *O*' *Z*' axis. The distance between the center of the cell and the axis of the coil was *C*.

Figure 1 shows the model representation of the cell membrane and the internal organelle, and their orientation to the coil that generates the magnetic field. Two coordinate systems were utilized to represent the cell and the coil, respectively.

The co-centric spherical cell and the organelle were represented in a spherical coordinate system (*r*, *θ*, *ϕ*) centered at point *O*. The cell membrane was represented as a very thin shell with inner radius *R*_-_, outer radius *R*_+ _and thickness *D*. The organelle membrane was represented as a very thin shell with inner radius *r*_-_, outer radius *r*_+ _and thickness *d*. The two membrane shells divided the cellular environment into five homogenous, isotropic regions: extracellular medium (0#), cytoplasm membrane (1#), intracellular cytoplasm (2#), organelle membrane (3#) and the organelle internal (4#). The dielectric permittivities and the conductivities in the five regions were *ε*_*i *_and *σ*_*i*_, respectively, where *i *represents the region number.

The low-frequency magnetic field was represented in a cylindrical coordinate system (*r*', *ϕ*', *z*'). The distance between the center of the cell (*O*) and the axis of the coil (*O*') was *C*. The externally applied, sinusoidally alternating magnetic field was symmetric about the *O*' *Z*' axis. The magnetic field was represented as , where  was the unit vector in the direction of *O*' *Z*', *ω *was the angular frequency of the magnetic field, and  was the imaginary unit.

### Model parameters

Table 1 lists the parameters used for the model. To quantitatively investigate the amount of polarization on both the cytoplasmic and organelle membranes, we chose their geometrical and electrical parameters (standard values, the lower and upper limits) from the literature [41]. The frequency range of interest was determined to be between 2 - 200 kHz. The upper limit was determined by calculating the reciprocal value of the rising phase of a current pulse during peripheral nerve stimulation [42,43]. Most frequencies used in the experimental practices were lower than this value [44]. The intensity of the magnetic field was 2 Tesla from TMS practice. The standard frequency of the magnetic field was estimated to be 10 kHz, as the rising time of single pulses was ~100 *μs *during TMS. This yielded the peak value of *dB*/*dt *= 2 × 10^4^*T*/*s *[45].

**Table 1:** Model parameters.

Parameters	Standard value	Lower limit	Upper limit
Extracellular conductivity (*σ*_0_, S/m)	1.2	-	-

Cell membrane conductivity (*σ*_1_, S/m)	3 × 10^-7^	1.0 × 10^-8^	1.0 × 10^-6^

Cytoplasmic conductivity (*σ*_2_, S/m)	0.3	0.1	1.0

Mitochondrion membrane conductivity (*σ*_3_, S/m)	3 × 10^-7^	1.0 × 10^-8^	1.0 × 10^-5^

Mitochondrion internal conductivity (*σ*_4_, S/m)	0.3	0.1	1.0

Extracellular dielectric permittivity (*ε*_0_, As/Vm)	6.4 × 10^-10^	-	-

Cell membrane dielectric permittivity (*ε*_1_, As/Vm)	4.4 × 10^-11^	1.8 × 10^-11^	8.8 × 10^-11^

Cytoplasmic dielectric permittivity (*ε*_2_, As/Vm)	6.4 × 10^-10^	3.5 × 10^-10^	7.0 × 10^-10^

Mitochondrion membrane permittivity (*ε*_3_, As/Vm)	4.4 × 10^-11^	1.8 × 10^-11^	8.8 × 10^-11^

Mitochondrion internal permittivity (*ε*_4_, As/Vm)	6.4 × 10^-10^	3.5 × 10^-10^	7.0 × 10^-10^

Cell radius (R, um)	10	5	100

Cell membrane thickness (*D*, *nm*)	5	3	7

Mitochondrion radius (r, um)	3	0.3	5

Mitochondrion membrane thickness (*d*, *nm*)	5	1	8

Magnetic field intensity (B_0_, Tesla)	2	-	-

Magnetic field frequency (*f*, *kHz*)	10	2	200

### Governing equations for potentials and electric fields induced by the time-varying magnetic field

The electric field induced by the time varying magnetic field in the biological media was(1)

where  is the magnetic vector potential induced by the current in the coil. The potential *V *was the electric scalar potential due to charge accumulation that appears from the application of a time-varying magnetic field [46]. In spherical coordinates (*r*, *θ*, *ϕ*), . Using quasi-static approximations, in charge-free regions, *V *was obtained by solving Laplace's equation(2)

### Boundary conditions

Four boundary conditions were considered in the derivation of the potentials induced by the time-varying magnetic field.

(A). The potential was continuous across the boundary of two different media. In this paper, this refers to the extracellular media/membrane interface (0#1#), the cell membrane/intracellular cytoplasm interface (1#2#), the intracellular cytoplasm/organelle membrane interface (2#3#), and the organelle membrane/organelle interior interface (3#4#).

(B). The normal component of the current density was continuous across two different media. For materials such as pure conductors, it was equal to the product of the electric field and the conductivity of the media. During time-varying field stimulation, the complex conductivity, defined as *S *= *σ *+*jωε*, was used to account for the dielectric permittivity of the material [47]. Here, *σ *was the conductivity, *ε *was the dielectric permittivity of the tissue, *ω *was the angular frequency of the field. Therefore, on the extracellular media/membrane interface (0#1#),(3)

On the cell membrane/intracellular cytoplasm interface (1#2#),(4)

On the intracellular cytoplasm/organelle membrane interface (2#3#),(5)

On the organelle membrane/organelle interior interface (3#4#),(6)

where *S*_0 _= *σ*_0_+*jωε*_0_, *S*_1 _= *σ*_1_+*jωε*_1_, *S*_2 _= *σ*_2_+*jωε*_2_, *S*_3 _= *σ*_3_+*jωε*_3 _and *S*_4 _= *σ*_4_+*jωε*_4 _were the complex conductivities of the five media, respectively.

(C). The electric field at an infinite distance from the cell was not perturbed by the presence of the cell.

(D). The potential inside the organelle (*r *= 0) was finite.

### Magnetic vector potential 

When the center of the magnetic field was at point O',  was in the direction of  since(7)

where vector potential  was in the direction of  (Figure 1). In cylindrical coordinates (*r*', *ϕ*', *z*'), the magnetic vector potential was expressed as (Appendix A in [19]):(8)

In order to calculate the potential distribution in the model cell, one needs to have an expression for  in spherical coordinates(*r*, *θ*, *ϕ*). By coordinate transformation (Appendix B in [19]), we obtained the magnetic vector potential  in spherical coordinates (*r*, *θ*, *ϕ*):(9)

The vector potential components in the , ,  directions were:(10)

### Software packages

Derivations of the equations were done with Mathematica 6.0 (Wolfram Research, Inc. Champaign, IL). Numerical simulations were done with Matlab 7.4.0 (The MathWorks, Inc. Natick, MA).

## Results

### Transmembrane potentials induced by a time-varying magnetic field

In spherical coordinates (*r*, *θ*, *ϕ*), the solution for Laplace's equation (2) can be written in the form(13)

where *C*_*n*_, *D*_*n *_were unknown coefficients (n = 0,1,2,3,4,5). We solved for those coefficients (Appendix) and substituted them into equation (13) to obtain the potential terms in the five model regions. Next, the transmembrane potential in a membrane can be obtained by subtracting the membrane potential at the inner surface from that at the outer surface.

In the cell membrane, the induced transmembrane potential was(14)

Where, .

In the organelle membrane, the induced transmembrane potential was(15)

Where,

Similar regional polarization patterns were observed between the cell membrane and the organelle membrane, since they both depended on a sin*θ*cos*ϕ *term. Since *θ *and *ϕ *were determined by the relative orientation of the coil to the cell, the patterns of polarization in the target cell and the organelle both depended on their orientations to the stimulation coil.

**Figure 2:**
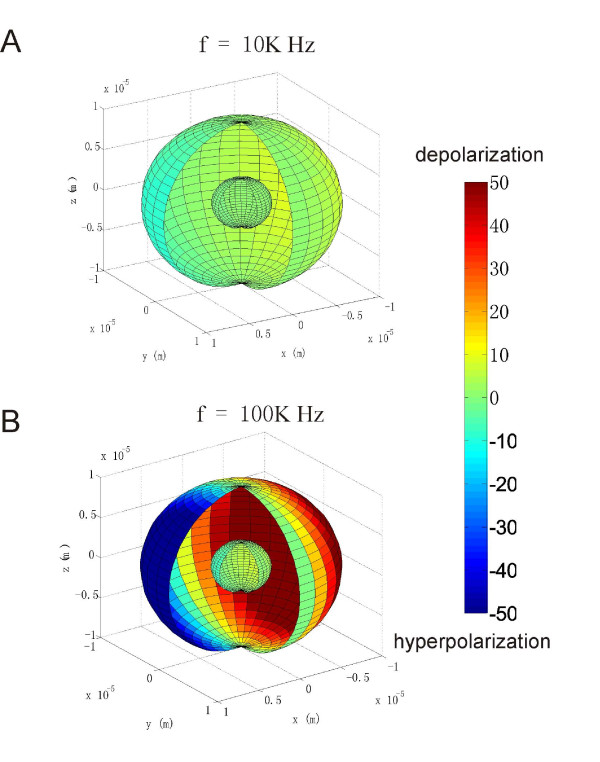
**Regional polarization of the cytoplasmic membrane and the organelle membrane by the time-varying magnetic field**. The plot demonstrated an instant polarization pattern on both membranes. A cleft was made to illustrate the internal structure. The orientation of the cell to the coil was the same as that shown in Figure 1B. The color map represented the amount of polarization (in mV) calculated with the standard values listed in table 1. A. Field frequency was 10 KHz. B. Field frequency was 100 KHz.

*ψ*_*cell *_and *ψ*_*org *_at one instant moment were plotted for 10 KHz and 100 KHz, respectively (Figure 2). The locations for the maximal polarization were on the equators of the cell and of the organelle membranes (*θ *= 90° or z = 0 plane). The two membranes were maximally depolarized at *ϕ *= 180° (deep red) and maximally hyperpolarized at *ϕ *= 0 (deep blue) on the equator, respectively. The cell and the organelle membranes were not polarized on the two poles corresponding to *θ *= 0° and *θ *= 180°, respectively. The full cycle of polarization by the time-varying magnetic field was also illustrated (see Additional file 1).

Both *ψ*_*cell *_and *ψ*_*org *_depended on the geometrical parameters of the cell (*R*_+_, *R*_-_, *C*) and the organelle (*r*_+_, *r*_-_), and the electrical properties of the five media considered in the model (*S*_0_, *S*_1_, *S*_2_, *S*_3_, *S*_4_). These parameters did not affect the polarization pattern. Therefore, we chose maximal polarizations (corresponding to the point that is defined by *θ *= 90°, *ϕ *= 270°) on the cell and organelle membranes (Figures 1 and 2) for the further analysis of their dependency on the field frequency.

### Frequency responses

Two factors contribute to the frequency-dependency of the polarizations (magnitude and phase) in the two membranes. First, the magnitude of the electrical field is proportional to the frequency of the externally applied magnetic field, as required by Faraday's law. Second, the dielectric properties of the material considered in the model are frequency-dependent.

**Figure 3:**
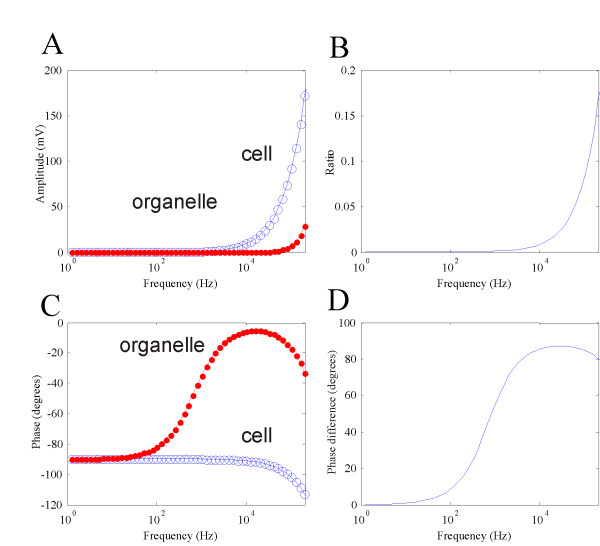
**The frequency dependency of *ψ*_*cell *_and *ψ*_*org*_**. A. Maximal amplitudes of *ψ*_*cell *_(large circle) and *ψ*_*org *_plotted as a function of field frequency. B. Ratio of the two membrane polarizations as a function of the field frequency. C. Phases of *ψ*_*cell *_(large circle) and *ψ*_*org *_plotted as a function of field frequency. D. Phase difference between the two membrane polarizations.

With the standard values, *ψ*_*cell *_was always greater than and *ψ*_*org *_(Figure 3A). At 10 kHz, the maximal polarization on the cell membrane was 9.397 mV, and the maximum polarization on the internal organelle was only 0.08 mV. Figure 3B plots the ratio of the two polarizations. As the frequency increased, *ψ*_*org *_became quantitatively comparable to *ψ*_*cell*_. At extremely high frequency (~100 MHz), the ratio reached a plateau of 1 (not shown).

The phase was defined as the phase difference between the externally applied magnetic field and membrane polarization, which was computed as the phase angle of the complex transmembrane potentials. Phase in the cell membrane was insensitive to the frequency change below 10 KHz. At 10 KHz, the phase in the cell membrane is -91.23°, which meant that an extra -1.23° was added to the membrane phase, due to frequency-dependent capacitive features of the tissue. On the other hand, phase response in the organelle membrane was more sensitive to the frequency change than the cell membrane, showing the dependence as low as 50 Hz. At 10 KHz, the phase in the organelle was -5.69°. Above 10 KHz, phases in both membranes increased with frequency. At 200 KHz, the phase in the cell membrane was -113.1°, and in the organelle membrane was -33.07°. Figure 3D plots the difference between the two phases as a function of frequency. At very low frequency (< 50 Hz), the two membranes demonstrated an in-phase polarization. At 10 KHz, their polarizations were nearly 90° out-of-phase.

### "Interaction" between the cell membrane and the organelle membrane

**Figure 4:**
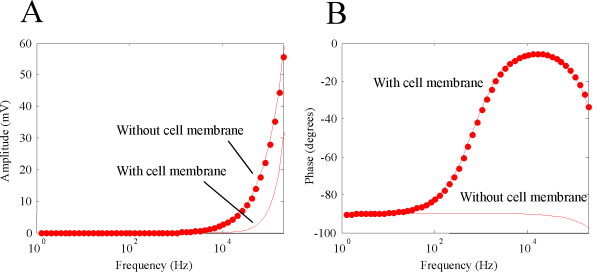
**"Shielding" effects of cytoplasmic membrane on the internal membrane**. A. Amplitude of *ψ*_*org *_with and without the presence of the cytoplasmic membrane. Presence of the cytoplasmic membrane reduced *ψ*_*org*_. B. Phase of *ψ*_*org *_with and without the presence of the cytoplasmic membrane.

Previous studies have shown that the cell membrane "shields" the internal cytoplasm and prevent the external field from penetrating inside the cell in electric stimulation [48,49]. Will similar phenomenon occur under magnetic stimulation? To estimate the impact of cell membrane on organelle polarization, we compared *ψ*_*org *_with and without the presence of the cell membrane. The later was done by letting *S*_1 _= *S*_0 _and *S*_2 _= *S*_0 _in equation (15), which removed the cell membrane,

Removal of the cell membrane allowed greater organelle polarization (Figure 4A). At 10 KHz, *ψ*_*org *_was 2.82 mV in the absence of the cell membrane, which was considerably greater than 0.08 mV for the case with the cell membrane. This screening effect was more prominent at 200 KHz, where *ψ*_*org *_was only 28.78 mV in the intact cell, and 55.87 mV without the cell membrane.

The phase response for the isolated organelle was similar to a cell membrane that was directly exposed in the field (Figure 4B). Therefore, presence of the cell membrane not only" shielded" the internal mitochondria from excessive polarization by the external field, but also provides an extra phase term that reduce the phase delay between the field and the organelle response.

**Figure 5:**
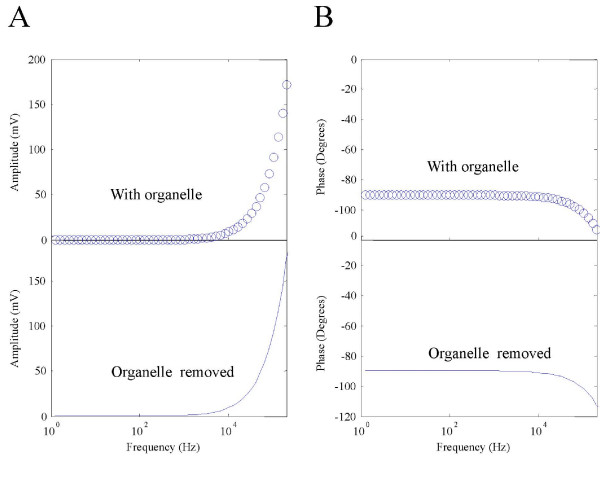
**Impact of the presence of internal organelle on *ψ*_*cell*_**. Amplitude (A) and phase (B) of *ψ*_*cell *_with the presence of the internal organelle (cycle) or after the organelle was removed from the cell (line).

Alteration in the organelle polarization by removing the cell membrane suggested an "interactive" effect between the two membranes via electric fields. We next asked if the presence of the internal organelle might have the reciprocal effects on *ψ*_*cell*_. To test this possibility, we removed the internal organelle and investigated its effect on *ψ*_*cell*_. This was done by letting *S*_3 _= *S*_2 _and *S*_4 _= *S*_2 _in equation (14). Removal of the internal organelle did not introduce significant changes on *ψ*_*cell *_(Figure 5). Removal of the organelle led to a 0.001 mV increase in *ψ*_*cell *_at 10 KHz, and a 1.3 mV increase at 200 KHz, respectively. The phase change caused by organelle removal was only 0.7 degrees at 200 KHz. These results suggest that the presence of the internal organelle only had trivial effects on the cytoplasmic membrane.

### Dependency of *ψ*_*org *_on the cell membrane parameters

**Figure 6:**
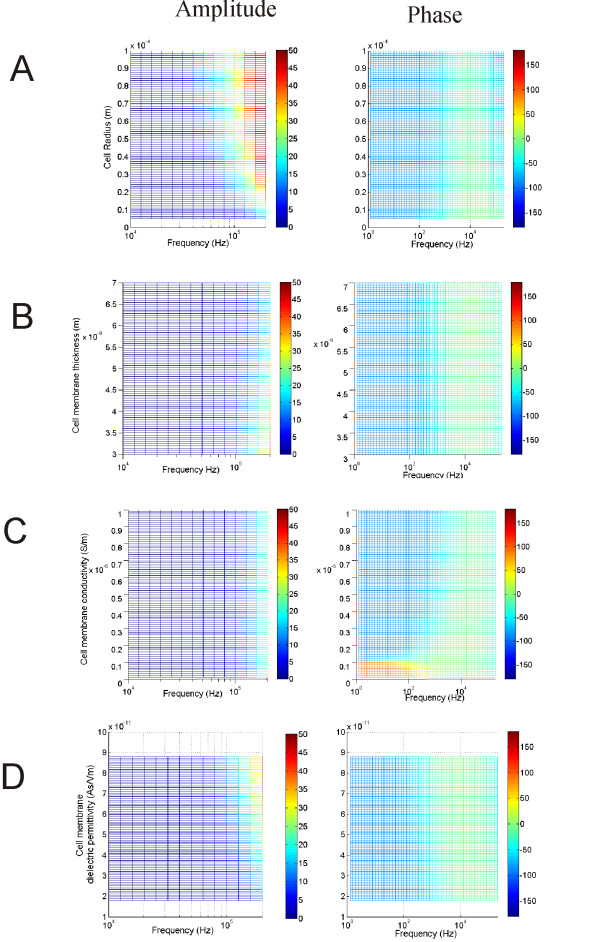
**Dependency of *ψ*_*org *_on the cytoplasmic membrane properties**. Effects of cell diameter (A), cell membrane thickness (B), cell membrane conductivity (C) and cell membrane di-electricity (D) on the amplitude and phase of *ψ*_*org*_.

To further investigate the shielding effects of the cell membrane on *ψ*_*org*_, we systemically varied the cell membrane parameters within their physiological ranges, and studied their individual impacts on the organelle polarization. These parameters included the geometrical properties (radius and membrane thickness) and the electrical properties (cell membrane conductivity and dielectric permittivity) of the cell membrane. This was done by varying one parameter through its given range but maintaining the others at their standard values. Since the dielectric properties of the tissues were frequency dependent, the parameter sweep was done within a frequency range (2 - 200 KHz). This generated a set of data that could be depicted in a color plot of *ψ*_*org *_(amplitude or phase) as a function of frequency and the studied parameters (Figures 6).

At a low frequency band (< 10 KHz), *ψ*_*org *_was trivial, since the magnitude of the induced electric field was small. *ψ*_*org *_became considerably large beyond 10 KHz. Increase in the cell radius facilitates this polarization (Figure 6A left). Increase in the cell radius did not significantly change the phase-frequency relation in the organelle. However, it increased the phase at relatively high frequency (~100 KHz, Figure 6A right). Increase in the cell membrane thickness compromised *ψ*_*org*_, so that higher frequency was needed to induce considerable polarization in the organelle (Figure 6B left). Variation in membrane thickness did not significantly alter the phase of the organelle polarization (Figure 6B right). Since removal of the low-conductive cell membrane enhanced organelle polarization (Figure 4A), one might expect that an increase in the membrane conductivity could have a similar effect. However, within the physiological range considered in this paper, *ψ*_*org *_was insensitive to the cell membrane conductivity (Figure 6C left). The cell membrane conductivity did have a significant impact on the phase of mitochondria polarization. At extremely low values (<10^-7^*S*/*m*), *ψ*_*org *_demonstrated a phase advance at frequency lower than 1 KHz (Figure 6C right), rather than a phase delay, as was the case for the standard values (Figure 3C). The cell membrane dielectric permittivity represents the capacitive property of the membrane. Increase in this parameter facilitated *ψ*_*org*_, so that *ψ*_*org *_became noticeable at relatively lower frequency range (Figure 6D left). An increase in this parameter also led to a decrease in the phase delay in the organelle polarization, which was most prominent at the frequency above 100 Hz (Figure 6D right).

### Dependency of *ψ*_*org *_on its own biophysics

Previous studies have shown that polarization of a neuronal structure depends on its own membrane properties under both electrical [48], and magnetic stimulations [19]. How do the membrane properties of the organelle membrane affect its own polarization?

**Figure 7:**
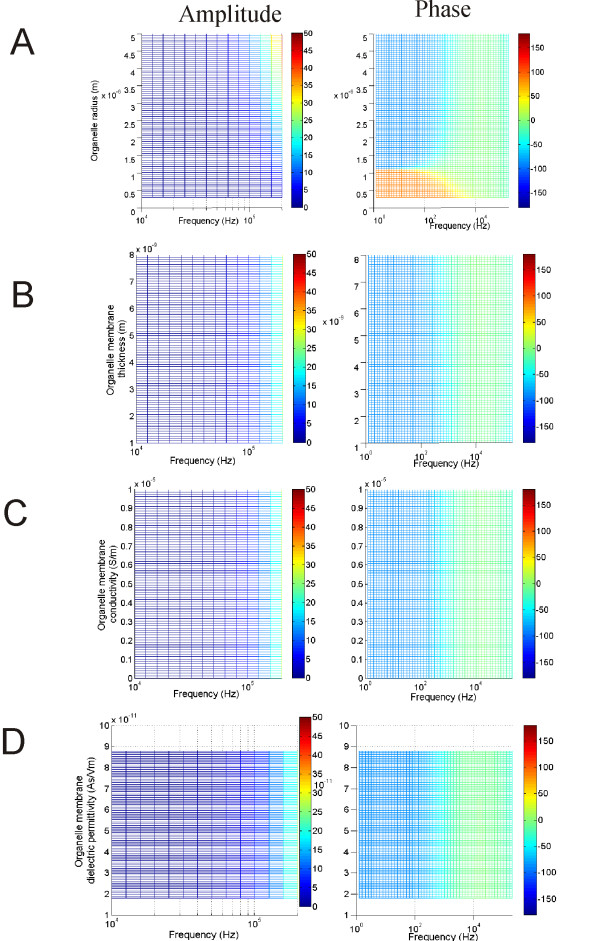
**Dependency of *ψ*_*org *_on its own membrane properties**. Effects of organelle diameter (A), thickness (B), membrane conductivity (C) and membrane di-electricity (D) on the amplitude and phase of *ψ*_*org*_.

An increase in the organelle radius led to a greater *ψ*_*org *_(Figure 7A, left). The phase-frequency relationship differentiated at a radius value around 1.1 um. Above this value, the phase response followed a pattern depicted in Figure 3C, i.e., the phase delay was -90 degree for low frequency and decreased to 0 at around 10 K Hz. Below this value, the phase showed a 90-degree advance instead of a lag in the low frequency range < 10 K Hz (Figure 7A, right). The membrane thickness has been generally agreed to be least significant to membrane polarization [50]. Varying membrane thickness in the organelle did not cause significant change in the magnitude (Figure 7B, left) nor the phase (Figure 7B, right) of *ψ*_*org*_. *ψ*_*org *_was also insensitive to its own electrical properties. Varying membrane conductivity (Figure 7C) or dielectricity (Figure 7D) in the organelle did not alter the frequency-dependent polarization in this structure.

## Discussion

### Similarities and differences to electrical stimulation

Analysis of *ψ*_*org *_under magnetic stimulation reveals several commonalities and differences to that under electric stimulation. The build up of *ψ*_*org *_requires the electric field to penetrate through the cytoplasmic membrane. In electric stimulation, this is achieved by directly applied electric current via electrodes. In magnetic stimulation, electric field is produced by electromagnetic induction.

Analysis on *ψ*_*org *_under electric field has been performed in two recent publications. Vajrala et al. [28] developed a three-membrane model that included the inner and our membranes of a mitochondrion, and have analytically solved *ψ*_*cell *_and *ψ*_*org *_under oscillatory electric fields. Another study [41] has modeled the internal membrane response to the time-varying electric field, and has investigated the condition under which *ψ*_*org *_can temporarily exceed *ψ*_*cell *_under nanosecond duration pulsed electric fields.

Results obtained from this magnetic study share several commonalities with those from AC electric stimulation. Under both stimulation conditions, *ψ*_*org *_can never exceed *ψ*_*cell*_. The ratio between the (organelle/cell) increases with frequency, and this ratio can reach 1 at very high frequency (10^8 ^Hz, data not shown). The phase responses of the organelle within a cell have not been analyzed previously under electric stimulation, which prevent direct comparison with this work. For an isolated mitochondrion, its response is similar to a single cell membrane under AC electric field stimulation [47], except that an extra -90° phase is introduced by electromagnetic induction (Figure 4B).

Stimulation on the internal organelle by time-varying magnetic field, though, has its own uniqueness. First, as a non-invasive method, magnetic stimulation is achieved by current induction inside the tissue, which prevents direct contact with the electrodes and introduces minimal discomfort. Second, the frequency responses of the internal organelle are different under the two stimulation protocols. In electric stimulation, magnitude of the field is independent of its frequency. In magnetic stimulation, however, the magnitude of the induced electric field is proportional to the frequency of the magnetic field (Faraday's law). Consequently, alteration in the field frequency could also contribute to *ψ*_*org*_. Low frequency field (< 1 KHz) is insufficient in building up noticeable *ψ*_*org *_and *ψ*_*cell *_(Figure 3A). Both *ψ*_*org *_and *ψ*_*cell *_increase with field frequency (Figure 3A). Therefore, it is unlikely possible to use high-frequency magnetic field to specifically target internal organelles, such as been done under AC electric stimulation with nanosecond pulses, for mitochondria electroporation and for the induction of mitochondria-dependent apoptosis [33].

### Cellular factors that influence *ψ*_*cell*_

When a neuron is exposed to an electric field, a transmembrane potential is induced on its membrane. Attempts to analytically solve *ψ*_*cell *_began as early as the 1950s [51,52]. Later works added more complexity to the modeled cell and provided insights into the factors affecting *ψ*_*cell*_. These include electrical properties [49,50,53,54] of the cell, such as its membrane conductivity. Geometrical properties of the cell could also affect *ψ*_*cell*_, such as its shape [55,56] and orientation to the field [57,58].

Presence of neighboring cells affect *ψ*_*cell *_in a tissue with high-density cells, For example, isthmo-optic cells in pigeons can be excited by electrical field effect through ephaptic interaction produced by the nearby cells whose axons were activated by electric stimulation, suggesting that electrical field effect may play important roles in interneuronal communications [59]. In infinite cell suspensions, *ψ*_*cell *_depended on cell volume fraction and cell arrangement [57]. Theoretical studies have proved that presence of a single cell affected *ψ*_*cell *_in its neighboring cells, without direct physical contact between the two cells [60].

This work investigates another important factor that might affect *ψ*_*cell*_, i.e., presence of the internal organelle. We have previously solved *ψ*_*cell *_for a spherical cell model under magnetic field stimulation, without considering the presence of the internal organelle [19]. This work extends the previous study by including an internal organelle in the cell model. Here, adding an organelle to the cell internal did not significantly change the magnitude and phase of *ψ*_*cell *_(Figure 5).

### Factors that influence *ψ*_*org *_during magnetic stimulation

Biological tissue is composed of many non-homogenous, anisotropic components, such as the cellular/axonal membrane, the internal organelles and the extracellular medium. The electrical properties (i.e., conductivities) of the tissue may vary with location in the tissue, even at a microscopic level. Under magnetic stimulation, several studies have provided insights into the impact of tissue properties on field distribution and tissue polarization [42,61].

This work further illustrates that the effects of magnetic stimulation are a function of tissue properties, by providing evidence that both the geometrical and electrical parameters of the cell/organelle membranes affect *ψ*_*org*_. Both the radius of the cell and the organelle strongly affect *ψ*_*org*_, which is in agree with previous studies [48,62]. Radius of the neuronal structure is important in determining the threshold for its own membrane polarization, as proved by in vitro studies on eukaryotic [63] and bacterial cells [64]. This model prediction is potentially testable with voltage-sensitive dyes that can provide both high temporal and high spatial resolutions [23,65]. Another model prediction is that the amount of *ψ*_*org *_is insensitive to the change in cell membrane conductivity. Evidence has shown that electric field can cause long-lasting increase in passive electrical conductance of the cell membrane, probably by opening of stable conductance pores [66]. The opening and closing of ion channels can also alter the membrane conductance. This model prediction can be tested by varying membrane conductivity, using ion-channel blockers applied to the cell membrane.

### Implications for transcranial magnetic stimulation (TMS)

Another important finding in this study that within the frequency band used TMS, *ψ*_*org *_is insignificant comparing with *ψ*_*cell*_. At 10 KHz, a frequency that corresponds to the rising time of the electric pulses used in clinical TMS, the field causes considerable amount of change in *ψ*_*cell*_, but only 0.08 mV change in *ψ*_*org *_(Figure 3A). It is worth noting that even this value was probably a consequence of overestimation in the magnetic field intensity (*B*_0_). To simplify the calculation, B0 was a constant (2 Tesla) everywhere in the modeled region. In reality, the intensity of the magnetic field generated by a coil could decay quickly in the tissue far away from the coil [67,68]. The duration of the stimulation time was also likely overestimated. During TMS, neuronal responses are induced by pulses, as opposed to the mathematically more tractable sinusoidal stimulus used in this model. Under this scenario, the magnetically-induced electric field in the tissue (essentially the change in the transmembrane potential) is determined by , which means the transmembrane potential can only be induced during the rise time (and decay time) during a step in the B field. Indeed, rise times of the field affect stimulation in clinic practice, and a faster rise time pulse is more efficient [45]. Therefore, *ψ*_*org *_is unlikely significant enough in TMS to have physiological implications, and internal organelles such as mitochondria are not likely be the target in TMS practice. This conclusion is made after extensive analysis on model parameters with the values in broad physiological ranges (Table 1). To our knowledge and based on a Medline search, there have been no reports on mitochondria-related effects in TMS practice.

This paper provides two mechanisms to account for the ineffectiveness of magnetically-induced polarization in internal organelles under TMS parameters. First, the cell membrane, which is made up of lipids and proteins, provides a dominant "shielding effect" on the organelles and prevents certain amount of electric fields to penetrate into the cell membrane and polarize the organelle membrane (Figure 4). Second, the radius of the organelle is always much smaller than that of the cell, which render them relatively insensitive to the magnetic field.

### Future directions

Several simplifying assumptions were proposed in this model to facilitate the derivation of the analytical solutions. The model assumed that the cell was located in an electrically homogenous extracellular medium, which was an over-simplification of the true electrically anisotropic extracellular environment. Both the extracellular medium and cytoplasmic environment are not truly homogenous [69,70]. We found that neither parameter significantly affects the organelle or cytoplasmic membrane polarization (not shown).

Both the cell membrane and the mitochondria membranes were modeled as a single spherical shell. In reality, however, cellular structures have irregular shape, which may play an important role in the dynamics of membrane polarization [71,72]. The interior sphere was centered inside the cell to allow for mathematical simplicity of the model. However, as organelle locations vary spatially in a cell, we hypothesize that organelles located off-center of the cell or closer to the exterior cell membrane may be more sensitive to the applied field. Also, we believe the "shielding effect" of the cell membrane persists even when the separation distance between the two membranes is small (data not shown). The membrane of the organelle was modeled as a single internal shell as in a previous study [41], rather than a two-shell structure, representative of the inner and our membranes of a mitochondrion, respectively [28]. The highly curved projections of the cell body and the organelle membrane may provide focal points for even greater changes in the induced transmembrane potential [73]. Future study will use numerical methods with multi-compartment modeling or finite element meshes to represent these structure complexities.

All the dielectric permittivities in the model were assumed to be frequency-independent, which was valid for the low frequencies considered (10-200 kHz). When field frequency exceeds several hundreds of megahertz, the finite mobility of molecular dipoles starts to weaken the polarization processes [41]. This phenomenon, known as dielectric relaxation, is characterized with decrease in the permittivities and increase in the conductivity. When this happens, the complex conductivity should be defined as *S *= *σ *(*ω*) + *jωε *(*ω*), where *σ *(*ω*) and *ε *(*ω*) are frequency-dependent conductivity and permittivity, respectively. By implementing this term in equations (14) and (15), one can estimate the transmembrane potentials in the cell and in the organelle when dielectric relaxation occurs.

## Conclusions

This work provides the first analytical solution for the transmembrane potentials in an internal organelle (*ψ*_*org*_) in response to time-varying magnetic stimulation. The frequency response of the membrane under magnetic stimulation is different from that under electric field stimulation. This work provides evidence that the presence of the internal organelle does not significantly affect polarization of the cell membrane (*ψ*_*cell*_). Moreover, *ψ*_*org *_is always smaller than *ψ*_*cell *_under low frequency range (< 200 KHz), largely due to the "shielding effect" imposed by the presence of the cell membrane. Both the geometrical and electrical properties of the cell membrane affect *ψ*_*org *_in a frequency-dependent manner. The properties of the organelle membrane also affect *ψ*_*org *_in a frequency-dependent manner. Finally, the present study provides evidence that normal mitochondrial functionality is not likely affected by transcranial magnetic stimulation, via altering its membrane potential.

## Competing interests

The authors declare that they have no competing interests.

## Authors' contributions

HY was involved with model equation derivation, data analysis, and drafting of the manuscript. MC was involved in generating figures. MGF and PLC supervised and coordinated the study. In addition, MC, EEK, MGF and PLC helped in drafting of the manuscript. All authors read and approved the final manuscript.

## Appendix

Determining unknown coefficients C_*n*_, D_*n *_in equation (13) using boundary conditions

Since *V *was bounded at *r *= 0 and *r *→ ∞, from equation (13) we had

Therefore, expressions for the potential distribution in the extracellular media, the cell membrane, the cytoplasm, the organelle membrane, and organelle interior are:(A-1)

We substituted A_0*r *_(equation 10) and the  components of ∇*V *in the five regions into (1) to yield the expressions of the normal components of the electric fields in the five regions:(A-6)

Following boundary condition (A), *V *was continuous at the extracellular media/membrane (*r *= *R*_+_), the membrane/intracellular cytoplasm interfaces (*r *= *R*_-_), the cytoplasm/organelle interface and the organelle membrane/organelle interior interface.(A-11)

We then used the boundary condition (B), that the normal components of the current densities were continuous between two different media (equations 3-6), to obtain the following equations:(A-15)

We solved (A-11) to (A-18) the last eight unknown coefficients D_0_-D_3_, C_1_-C_4_. (see Additional file 2).

## Supplementary Material

Additional file 1 **Dynamic membrane potential changes in the cell and in the internal organelle**. A movie that shows the membrane potentials in the cell and in the organelle, induced by a 100 KHz magnetic field. Click here for file

Additional file 2 **Membrane potentials in the cell and in the internal organelle**. Mathematic derivations of the membrane potentials. Click here for file
