# Supplementary material for: Transmembrane potential induced on the internal organelle by a time-varying magnetic field: a model study
Source: J Neuroeng Rehabil. 2010 Feb 20;7:12. doi: 10.1186/1743-0003-7-12 (PMC2836366; doi:10.1186/1743-0003-7-12)
Supplement: Additional file 2 — Membrane potentials in the cell and in the internal organelle. Mathematic derivations of the membrane potentials. [file 1743-0003-7-12-S2.pdf]

1. Equations A – 11 to A – 14, as defined by boundary conditions (A) in the Appendix.

```
D0 / Rp^2 == C1 * Rp + D1 / Rp^2

C1 * Rm + D1 / Rm^2 == C2 * Rm + D2 / Rm^2

C2 * rp + D2 / rp^2 == C3 * rp + D3 / rp^2

C3 * rm + D3 / rm^2 == C4 * rm
```

2. Equations A – 15 to A – 18, as defined by boundary conditions (B) in the Appendix.

```
S0 * (M + 2 * D0 / Rp^3) == S1 * (M + 2 * D1 / Rp^3 - C1)

S1 * (M + 2 * D1 / Rm^3 - C1) == S2 * (M + 2 * D2 / Rm^3 - C2)

S2 * (M + 2 * D2 / rp^3 - C2) == S3 * (M + 2 * D3 / rp^3 - C3)

S3 * (M + 2 * D3 / rm^3 - C3) == S4 * (M - C4)
```

3. Solve equations A – 11 to A – 18 to obtain the unknown coefficients D0 – D3 and C1 – C4.

```
Solve[{D0 / Rp^2 == C1 * Rp + D1 / Rp^2,
  C1 * Rm + D1 / Rm^2 == C2 * Rm + D2 / Rm^2,
  C2 * rp + D2 / rp^2 == C3 * rp + D3 / rp^2,
  C3 * rm + D3 / rm^2 == C4 * rm,
  S0 * (M + 2 * D0 / Rp^3) == S1 * (M + 2 * D1 / Rp^3 - C1),
  S1 * (M + 2 * D1 / Rm^3 - C1) == S2 * (M + 2 * D2 / Rm^3 - C2),
  S2 * (M + 2 * D2 / rp^3 - C2) == S3 * (M + 2 * D3 / rp^3 - C3),
  S3 * (M + 2 * D3 / rm^3 - C3) == S4 * (M - C4)},
 {D0, C1, D1, C2, D2, C3, D3, C4}]
```

D0

$$\begin{aligned}&\Big(M\,Rp^3\,\Big(-rm^3\,\big(2\,Rm^6\,(S0+2\,S1)\,(S1-S2)\,(S2-S3)+2\,rp^3\,Rp^3\,(S0-S1)\,(S1-S2)\,(S2+2\,S3)+\\&\quad Rm^3\,\big(2\,Rp^3\,(S0-S1)\,(2\,S1+S2)\,(S2-S3)+rp^3\,(S0+2\,S1)\,(S1+2\,S2)\,(S2+2\,S3)\big)\big)\,(S3-S4)-\\&\quad rp^3\,\big(2\,rp^3\,Rp^3\,(S0-S1)\,(S1-S2)\,(S2-S3)+Rm^6\,(S0+2\,S1)\,(S1-S2)\,(2\,S2+S3)+\\&\quad Rm^3\,\big(rp^3\,(S0+2\,S1)\,(S1+2\,S2)\,(S2-S3)+Rp^3\,(S0-S1)\,(2\,S1+S2)\,(2\,S2+S3)\big)\big)\,(2\,S3+S4)\Big)\Big)\Big/\\&\Big(2\,rm^3\,\big(2\,Rm^6\,(S0-S1)\,(S1-S2)\,(S2-S3)+rp^3\,Rp^3\,(2\,S0+S1)\,(S1-S2)\,(S2+2\,S3)+\\&\quad Rm^3\,\big(Rp^3\,(2\,S0+S1)\,(2\,S1+S2)\,(S2-S3)+rp^3\,(S0-S1)\,(S1+2\,S2)\,(S2+2\,S3)\big)\big)\,(S3-S4)+\\&\quad rp^3\,\big(2\,rp^3\,Rp^3\,(2\,S0+S1)\,(S1-S2)\,(S2-S3)+2\,Rm^6\,(S0-S1)\,(S1-S2)\,(2\,S2+S3)+\\&\quad Rm^3\,\big(2\,rp^3\,(S0-S1)\,(S1+2\,S2)\,(S2-S3)+Rp^3\,(2\,S0+S1)\,(2\,S1+S2)\,(2\,S2+S3)\big)\big)\,(2\,S3+S4)\Big)\end{aligned}$$

C1

$$\begin{aligned}&\Big(M\,(S0-S1)\,\Big(2\,rm^3\,\big(2\,Rm^6\,(S1-S2)\,(S2-S3)-rp^3\,Rp^3\,(S1-S2)\,(S2+2\,S3)+\\&\quad Rm^3\,\big(-Rp^3\,(2\,S1+S2)\,(S2-S3)+rp^3\,(S1+2\,S2)\,(S2+2\,S3)\big)\big)\,(S3-S4)+\\&\quad rp^3\,\big(-2\,rp^3\,Rp^3\,(S1-S2)\,(S2-S3)+2\,Rm^6\,(S1-S2)\,(2\,S2+S3)+\\&\quad Rm^3\,\big(2\,rp^3\,(S1+2\,S2)\,(S2-S3)-Rp^3\,(2\,S1+S2)\,(2\,S2+S3)\big)\big)\,(2\,S3+S4)\Big)\Big)\Big/\\&\Big(2\,rm^3\,\big(2\,Rm^6\,(S0-S1)\,(S1-S2)\,(S2-S3)+rp^3\,Rp^3\,(2\,S0+S1)\,(S1-S2)\,(S2+2\,S3)+\\&\quad Rm^3\,\big(Rp^3\,(2\,S0+S1)\,(2\,S1+S2)\,(S2-S3)+rp^3\,(S0-S1)\,(S1+2\,S2)\,(S2+2\,S3)\big)\big)\,(S3-S4)+\\&\quad rp^3\,\big(2\,rp^3\,Rp^3\,(2\,S0+S1)\,(S1-S2)\,(S2-S3)+2\,Rm^6\,(S0-S1)\,(S1-S2)\,(2\,S2+S3)+\\&\quad Rm^3\,\big(2\,rp^3\,(S0-S1)\,(S1+2\,S2)\,(S2-S3)+Rp^3\,(2\,S0+S1)\,(2\,S1+S2)\,(2\,S2+S3)\big)\big)\,(2\,S3+S4)\Big)\end{aligned}$$

Printed by Mathematica for Students

D1

$$
\begin{aligned}
&-\Big(3\,M\,Rm^3\,Rp^3\,S0\,\big(rm^3\,\big(2\,Rm^3\,(S1-S2)\,(S2-S3)+rp^3\,(S1+2\,S2)\,(S2+2\,S3)\big)\,(S3-S4)+\\
&\quad rp^3\,\big(rp^3\,(S1+2\,S2)\,(S2-S3)+Rm^3\,(S1-S2)\,(2\,S2+S3)\big)\,(2\,S3+S4)\big)\Big)\Big/\\
&\Big(2\,rm^3\,\big(2\,Rm^6\,(S0-S1)\,(S1-S2)\,(S2-S3)+rp^3\,Rp^3\,(2\,S0+S1)\,(S1-S2)\,(S2+2\,S3)+\\
&\quad Rm^3\,\big(Rp^3\,(2\,S0+S1)\,(2\,S1+S2)\,(S2-S3)+rp^3\,(S0-S1)\,(S1+2\,S2)\,(S2+2\,S3)\big)\big)\,(S3-S4)+\\
&\quad rp^3\,\big(2\,rp^3\,Rp^3\,(2\,S0+S1)\,(S1-S2)\,(S2-S3)+2\,Rm^6\,(S0-S1)\,(S1-S2)\,(2\,S2+S3)+\\
&\quad Rm^3\,\big(2\,rp^3\,(S0-S1)\,(S1+2\,S2)\,(S2-S3)+Rp^3\,(2\,S0+S1)\,(2\,S1+S2)\,(2\,S2+S3)\big)\big)\,(2\,S3+S4)\Big)
\end{aligned}
$$

C2

$$
\begin{aligned}
&\Big(M\,\big(2\,rm^3\,\big(2\,Rm^6\,(S0-S1)\,(S1-S2)\,(S2-S3)+rp^3\,Rp^3\,(2\,S0+S1)\,(S1-S2)\,(S2+2\,S3)+\\
&\quad Rm^3\,\big(-Rp^3\,(S0\,(5\,S1-2\,S2)-S1\,(2\,S1+S2))\,(S2-S3)+rp^3\,(S0-S1)\,(S1+2\,S2)\,(S2+2\,S3)\big)\big)\\
&\quad (S3-S4)+rp^3\,\big(2\,rp^3\,Rp^3\,(2\,S0+S1)\,(S1-S2)\,(S2-S3)+2\,Rm^6\,(S0-S1)\,(S1-S2)\,(2\,S2+S3)+\\
&\quad Rm^3\,\big(2\,rp^3\,(S0-S1)\,(S1+2\,S2)\,(S2-S3)-Rp^3\,(S0\,(5\,S1-2\,S2)-S1\,(2\,S1+S2))\,(2\,S2+S3)\big)\big)\\
&\quad (2\,S3+S4)\big)\Big)\Big/\Big(2\,rm^3\,\big(2\,Rm^6\,(S0-S1)\,(S1-S2)\,(S2-S3)+rp^3\,Rp^3\,(2\,S0+S1)\,(S1-S2)\,(S2+2\,S3)+\\
&\quad Rm^3\,\big(Rp^3\,(2\,S0+S1)\,(2\,S1+S2)\,(S2-S3)+rp^3\,(S0-S1)\,(S1+2\,S2)\,(S2+2\,S3)\big)\big)\,(S3-S4)+\\
&\quad rp^3\,\big(2\,rp^3\,Rp^3\,(2\,S0+S1)\,(S1-S2)\,(S2-S3)+2\,Rm^6\,(S0-S1)\,(S1-S2)\,(2\,S2+S3)+\\
&\quad Rm^3\,\big(2\,rp^3\,(S0-S1)\,(S1+2\,S2)\,(S2-S3)+Rp^3\,(2\,S0+S1)\,(2\,S1+S2)\,(2\,S2+S3)\big)\big)\,(2\,S3+S4)\Big)
\end{aligned}
$$

D2

$$
\begin{aligned}
&-\Big(9\,M\,Rm^3\,rp^3\,Rp^3\,S0\,S1\,\big(rm^3\,(S2+2\,S3)\,(S3-S4)+rp^3\,(S2-S3)\,(2\,S3+S4)\big)\Big)\Big/\\
&\Big(2\,rm^3\,\big(2\,Rm^6\,(S0-S1)\,(S1-S2)\,(S2-S3)+rp^3\,Rp^3\,(2\,S0+S1)\,(S1-S2)\,(S2+2\,S3)+\\
&\quad Rm^3\,\big(Rp^3\,(2\,S0+S1)\,(2\,S1+S2)\,(S2-S3)+rp^3\,(S0-S1)\,(S1+2\,S2)\,(S2+2\,S3)\big)\big)\,(S3-S4)+\\
&\quad rp^3\,\big(2\,rp^3\,Rp^3\,(2\,S0+S1)\,(S1-S2)\,(S2-S3)+2\,Rm^6\,(S0-S1)\,(S1-S2)\,(2\,S2+S3)+\\
&\quad Rm^3\,\big(2\,rp^3\,(S0-S1)\,(S1+2\,S2)\,(S2-S3)+Rp^3\,(2\,S0+S1)\,(2\,S1+S2)\,(2\,S2+S3)\big)\big)\,(2\,S3+S4)\Big)
\end{aligned}
$$

C3

$$
\begin{aligned}
&\Big(M\,\big(2\,rm^3\,\big(2\,Rm^6\,(S0-S1)\,(S1-S2)\,(S2-S3)+rp^3\,Rp^3\,(2\,S0+S1)\,(S1-S2)\,(S2+2\,S3)+\\
&\quad Rm^3\,\big(Rp^3\,(2\,S0+S1)\,(2\,S1+S2)\,(S2-S3)+rp^3\,(S0-S1)\,(S1+2\,S2)\,(S2+2\,S3)\big)\big)\,(S3-S4)+\\
&\quad rp^3\,\big(2\,rp^3\,Rp^3\,(2\,S0+S1)\,(S1-S2)\,(S2-S3)+2\,Rm^6\,(S0-S1)\,(S1-S2)\,(2\,S2+S3)+\\
&\quad Rm^3\,\big(2\,rp^3\,(S0-S1)\,(S1+2\,S2)\,(S2-S3)+\\
&\quad Rp^3\,(S1\,(2\,S1+S2)\,(2\,S2+S3)+S0\,(2\,S2\,(2\,S2+S3)+S1\,(-19\,S2+4\,S3)))\big)\big)\,(2\,S3+S4)\big)\Big)\Big/\\
&\Big(2\,rm^3\,\big(2\,Rm^6\,(S0-S1)\,(S1-S2)\,(S2-S3)+rp^3\,Rp^3\,(2\,S0+S1)\,(S1-S2)\,(S2+2\,S3)+\\
&\quad Rm^3\,\big(Rp^3\,(2\,S0+S1)\,(2\,S1+S2)\,(S2-S3)+rp^3\,(S0-S1)\,(S1+2\,S2)\,(S2+2\,S3)\big)\big)\,(S3-S4)+\\
&\quad rp^3\,\big(2\,rp^3\,Rp^3\,(2\,S0+S1)\,(S1-S2)\,(S2-S3)+2\,Rm^6\,(S0-S1)\,(S1-S2)\,(2\,S2+S3)+\\
&\quad Rm^3\,\big(2\,rp^3\,(S0-S1)\,(S1+2\,S2)\,(S2-S3)+Rp^3\,(2\,S0+S1)\,(2\,S1+S2)\,(2\,S2+S3)\big)\big)\,(2\,S3+S4)\Big)
\end{aligned}
$$

D3

$$
\begin{aligned}
&\Big(27\,M\,rm^3\,Rm^3\,rp^3\,Rp^3\,S0\,S1\,S2\,(-S3+S4)\Big)\Big/\\
&\Big(2\,rm^3\,\big(2\,Rm^6\,(S0-S1)\,(S1-S2)\,(S2-S3)+rp^3\,Rp^3\,(2\,S0+S1)\,(S1-S2)\,(S2+2\,S3)+\\
&\quad Rm^3\,\big(Rp^3\,(2\,S0+S1)\,(2\,S1+S2)\,(S2-S3)+rp^3\,(S0-S1)\,(S1+2\,S2)\,(S2+2\,S3)\big)\big)\,(S3-S4)+\\
&\quad rp^3\,\big(2\,rp^3\,Rp^3\,(2\,S0+S1)\,(S1-S2)\,(S2-S3)+2\,Rm^6\,(S0-S1)\,(S1-S2)\,(2\,S2+S3)+\\
&\quad Rm^3\,\big(2\,rp^3\,(S0-S1)\,(S1+2\,S2)\,(S2-S3)+Rp^3\,(2\,S0+S1)\,(2\,S1+S2)\,(2\,S2+S3)\big)\big)\,(2\,S3+S4)\Big)
\end{aligned}
$$

```
C4
```

$$
\begin{aligned}
&(M\,(2\,rm^3\,(2\,Rm^6\,(S0-S1)\,(S1-S2)\,(S2-S3)+rp^3\,Rp^3\,(2\,S0+S1)\,(S1-S2)\,(S2+2\,S3)+\\
&\quad Rm^3\,(Rp^3\,(2\,S0+S1)\,(2\,S1+S2)\,(S2-S3)+rp^3\,(S0-S1)\,(S1+2\,S2)\,(S2+2\,S3)))\,(S3-S4)+\\
&\quad rp^3\,(2\,rp^3\,Rp^3\,(2\,S0+S1)\,(S1-S2)\,(S2-S3)\,(2\,S3+S4)+\\
&\quad 2\,Rm^6\,(S0-S1)\,(S1-S2)\,(2\,S2+S3)\,(2\,S3+S4)+\\
&\quad Rm^3\,(2\,rp^3\,(S0-S1)\,(S1+2\,S2)\,(S2-S3)\,(2\,S3+S4)+Rp^3\,(S1\,(2\,S1+S2)\,(2\,S2+S3)\,(2\,S3+S4)+\\
&\quad S0\,(2\,S2\,(2\,S2+S3)\,(2\,S3+S4)+S1\,(-65\,S2\,S3+8\,S3^2+8\,S2\,S4+4\,S3\,S4)))))))\,/\\
&(2\,rm^3\,(2\,Rm^6\,(S0-S1)\,(S1-S2)\,(S2-S3)+rp^3\,Rp^3\,(2\,S0+S1)\,(S1-S2)\,(S2+2\,S3)+\\
&\quad Rm^3\,(Rp^3\,(2\,S0+S1)\,(2\,S1+S2)\,(S2-S3)+rp^3\,(S0-S1)\,(S1+2\,S2)\,(S2+2\,S3)))\,(S3-S4)+\\
&\quad rp^3\,(2\,rp^3\,Rp^3\,(2\,S0+S1)\,(S1-S2)\,(S2-S3)+2\,Rm^6\,(S0-S1)\,(S1-S2)\,(2\,S2+S3)+\\
&\quad Rm^3\,(2\,rp^3\,(S0-S1)\,(S1+2\,S2)\,(S2-S3)+Rp^3\,(2\,S0+S1)\,(2\,S1+S2)\,(2\,S2+S3)))\,(2\,S3+S4))
\end{aligned}
$$

4. Transmembrane potential on the cytoplasmic membrane

```
V1 = C1 * xx + D1 / xx^2
```

```
Vm = (C1 * Rm + D1 / Rm^2) - (C1 * Rp + D1 / Rp^2)
```

```
Simplify[Vm]
```

$$
\begin{aligned}
&(M\,(3\,Rm^3\,Rp\,S0\,(rm^3\,(2\,Rm^3\,(S1-S2)\,(S2-S3)+rp^3\,(S1+2\,S2)\,(S2+2\,S3))\,(S3-S4)+\\
&\quad rp^3\,(rp^3\,(S1+2\,S2)\,(S2-S3)+Rm^3\,(S1-S2)\,(2\,S2+S3))\,(2\,S3+S4))-\\
&\quad 3\,Rm\,Rp^3\,S0\,(rm^3\,(2\,Rm^3\,(S1-S2)\,(S2-S3)+rp^3\,(S1+2\,S2)\,(S2+2\,S3))\,(S3-S4)+\\
&\quad rp^3\,(rp^3\,(S1+2\,S2)\,(S2-S3)+Rm^3\,(S1-S2)\,(2\,S2+S3))\,(2\,S3+S4))+\\
&\quad Rm\,(S0-S1)\,(2\,rm^3\,(2\,Rm^6\,(S1-S2)\,(S2-S3)-rp^3\,Rp^3\,(S1-S2)\,(S2+2\,S3)+\\
&\quad Rm^3\,(-Rp^3\,(2\,S1+S2)\,(S2-S3)+rp^3\,(S1+2\,S2)\,(S2+2\,S3)))\,(S3-S4)+\\
&\quad rp^3\,(-2\,rp^3\,Rp^3\,(S1-S2)\,(S2-S3)+2\,Rm^6\,(S1-S2)\,(2\,S2+S3)+\\
&\quad Rm^3\,(2\,rp^3\,(S1+2\,S2)\,(S2-S3)-Rp^3\,(2\,S1+S2)\,(2\,S2+S3)))\,(2\,S3+S4))-\\
&\quad Rp\,(S0-S1)\,(2\,rm^3\,(2\,Rm^6\,(S1-S2)\,(S2-S3)-rp^3\,Rp^3\,(S1-S2)\,(S2+2\,S3)+\\
&\quad Rm^3\,(-Rp^3\,(2\,S1+S2)\,(S2-S3)+rp^3\,(S1+2\,S2)\,(S2+2\,S3)))\,(S3-S4)+\\
&\quad rp^3\,(-2\,rp^3\,Rp^3\,(S1-S2)\,(S2-S3)+2\,Rm^6\,(S1-S2)\,(2\,S2+S3)+\\
&\quad Rm^3\,(2\,rp^3\,(S1+2\,S2)\,(S2-S3)-Rp^3\,(2\,S1+S2)\,(2\,S2+S3)))\,(2\,S3+S4))))\,/\\
&(2\,rm^3\,(2\,Rm^6\,(S0-S1)\,(S1-S2)\,(S2-S3)+rp^3\,Rp^3\,(2\,S0+S1)\,(S1-S2)\,(S2+2\,S3)+\\
&\quad Rm^3\,(Rp^3\,(2\,S0+S1)\,(2\,S1+S2)\,(S2-S3)+rp^3\,(S0-S1)\,(S1+2\,S2)\,(S2+2\,S3)))\,(S3-S4)+\\
&\quad rp^3\,(2\,rp^3\,Rp^3\,(2\,S0+S1)\,(S1-S2)\,(S2-S3)+2\,Rm^6\,(S0-S1)\,(S1-S2)\,(2\,S2+S3)+\\
&\quad Rm^3\,(2\,rp^3\,(S0-S1)\,(S1+2\,S2)\,(S2-S3)+Rp^3\,(2\,S0+S1)\,(2\,S1+S2)\,(2\,S2+S3)))\,(2\,S3+S4))
\end{aligned}
$$

5. Transmembrane potential on the internal membrane

```
Vinternal = (C3 * rm + D3 / rm^2) - (C3 * rp + D3 / rp^2)
```

```
Simplify[Vinternal]
```

$$
\begin{aligned}
&\big(M\,\big(27\,rm^3\,Rm^3\,rp\,Rp^3\,S0\,S1\,S2\,(S3-S4)+27\,rm\,Rm^3\,rp^3\,Rp^3\,S0\,S1\,S2\,(-S3+S4)+\\
&\quad rm\,\big(2\,rm^3\,\big(2\,Rm^6\,(S0-S1)\,(S1-S2)\,(S2-S3)+rp^3\,Rp^3\,(2\,S0+S1)\,(S1-S2)\,(S2+2\,S3)+\\
&\qquad Rm^3\,\big(Rp^3\,(2\,S0+S1)\,(2\,S1+S2)\,(S2-S3)+rp^3\,(S0-S1)\,(S1+2\,S2)\,(S2+2\,S3)\big)\big)\,(S3-S4)+\\
&\quad rp^3\,\big(2\,rp^3\,Rp^3\,(2\,S0+S1)\,(S1-S2)\,(S2-S3)+2\,Rm^6\,(S0-S1)\,(S1-S2)\,(2\,S2+S3)+\\
&\qquad Rm^3\,\big(2\,rp^3\,(S0-S1)\,(S1+2\,S2)\,(S2-S3)+\\
&\qquad\quad Rp^3\,(S1\,(2\,S1+S2)\,(2\,S2+S3)+S0\,(2\,S2\,(2\,S2+S3)+S1\,(-19\,S2+4\,S3)))\big)\big)\,(2\,S3+S4)\big)-\\
&\quad rp\,\big(2\,rm^3\,\big(2\,Rm^6\,(S0-S1)\,(S1-S2)\,(S2-S3)+rp^3\,Rp^3\,(2\,S0+S1)\,(S1-S2)\,(S2+2\,S3)+\\
&\qquad Rm^3\,\big(Rp^3\,(2\,S0+S1)\,(2\,S1+S2)\,(S2-S3)+rp^3\,(S0-S1)\,(S1+2\,S2)\,(S2+2\,S3)\big)\big)\,(S3-S4)+\\
&\quad rp^3\,\big(2\,rp^3\,Rp^3\,(2\,S0+S1)\,(S1-S2)\,(S2-S3)+2\,Rm^6\,(S0-S1)\,(S1-S2)\,(2\,S2+S3)+\\
&\qquad Rm^3\,\big(2\,rp^3\,(S0-S1)\,(S1+2\,S2)\,(S2-S3)+Rp^3\,(S1\,(2\,S1+S2)\,(2\,S2+S3)+\\
&\qquad\quad S0\,(2\,S2\,(2\,S2+S3)+S1\,(-19\,S2+4\,S3)))\big)\big)\,(2\,S3+S4)\big)\big)\big)\Big/\\
&\big(2\,rm^3\,\big(2\,Rm^6\,(S0-S1)\,(S1-S2)\,(S2-S3)+rp^3\,Rp^3\,(2\,S0+S1)\,(S1-S2)\,(S2+2\,S3)+\\
&\quad Rm^3\,\big(Rp^3\,(2\,S0+S1)\,(2\,S1+S2)\,(S2-S3)+rp^3\,(S0-S1)\,(S1+2\,S2)\,(S2+2\,S3)\big)\big)\,(S3-S4)+\\
&\quad rp^3\,\big(2\,rp^3\,Rp^3\,(2\,S0+S1)\,(S1-S2)\,(S2-S3)+2\,Rm^6\,(S0-S1)\,(S1-S2)\,(2\,S2+S3)+\\
&\quad Rm^3\,\big(2\,rp^3\,(S0-S1)\,(S1+2\,S2)\,(S2-S3)+Rp^3\,(2\,S0+S1)\,(2\,S1+S2)\,(2\,S2+S3)\big)\big)\,(2\,S3+S4)\big)
\end{aligned}
$$

Printed by Mathematica for Students
